# Tensile strength of adhesives in peripheral nerve anastomoses: an in vitro biomechanical evaluation of four different neurorrhaphies

**DOI:** 10.1186/s40001-024-01858-9

**Published:** 2024-05-03

**Authors:** Marius Heitzer, Konrad Kilic, Ricarda Merfort, Philipp Winnand, Caroline Emonts, Anna Bock, Mark Ooms, Timm Steiner, Frank Hölzle, Ali Modabber

**Affiliations:** 1 Department of Oral and Maxillofacial Surgery, University Hospital of RWTH Aachen, Pauwelsstraße 13, 52074 Aachen, Germany; 2 Department of Orthopedics, Trauma and Reconstructive Surgery, University Hospital of RWTH Aachen, Aachen, Germany; 3 Institute of Textile Technology, RWTH Aachen University, Otto-Blumenthal-Straße 1, 52074 Aachen, Germany

**Keywords:** Tissue adhesives, Microsurgery, Polyurethanes, Tensile strength, Anastomosis

## Abstract

**Background:**

The fundamental prerequisite for prognostically favorable postoperative results of peripheral nerve repair is stable neurorrhaphy without interruption and gap formation.

**Methods:**

This study evaluates 60 neurorrhaphies on femoral chicken nerves in terms of the procedure and the biomechanical properties. Sutured neurorrhaphies (*n* = 15) served as control and three sutureless adhesive-based nerve repair techniques: Fibrin glue (*n* = 15), Histoacryl glue (*n* = 15), and the novel polyurethane adhesive VIVO (*n* = 15). Tensile and elongation tests of neurorrhaphies were performed on a tensile testing machine at a displacement rate of 20 mm/min until failure. The maximum tensile force and elongation were recorded.

**Results:**

All adhesive-based neurorrhaphies were significant faster in preparation compared to sutured anastomoses (*p* < 0.001). Neurorrhaphies by sutured (102.8 [cN]; *p* < 0.001), Histoacryl (91.5 [cN]; *p* < 0.001) and VIVO (45.47 [cN]; *p* < 0.05) withstood significant higher longitudinal tensile forces compared to fibrin glue (10.55 [cN]). VIVO, with △L/L_0_ of 6.96 [%], showed significantly higher elongation (*p* < 0.001) compared to neurorrhaphy using fibrin glue.

**Conclusion:**

Within the limitations of an in vitro study the adhesive-based neurorrhaphy technique with VIVO and Histoacryl have the biomechanical potential to offer alternatives to sutured neuroanastomosis because of their stability, and faster handling. Further in vivo studies are required to evaluate functional outcomes and confirm safety.

## Background

Facial palsy can be caused by various circumstances and is the most common cranial nerve lesion in clinical practice [1]. If the nerve lesion does not heal satisfactorily or chronic healing problems occur, surgical neurorrhaphy is often indicated [2]. Although there has been a multifaceted search for peripheral nerve repair techniques for over 80 years, there is still no absolute gold standard for anastomosis of severed nerves. Even with current technological advances, 15–50% of patients rate the postoperative outcome of neurorrhaphy as inadequate [3–5]. In addition to misalignment of the fascicles, an interruption or gap is a common factor in poor recovery after nerve repair [6]. A gap at the repair site in neurorrhaphy of the median nerve at the wrist or the radial nerve at the elbow poses challenging problems for surgeons due to a certain tension present at the repair site [6]. According to Grewal et al. this results from a normal in situ stress of a peripheral nerve [7]. Therefore, it is essential that the supply of a nerve repair ensures a high stability to withstand these physiological tension forces.

Historically, the use of microsurgical sutures has been the gold standard for peripheral nerve neurorrhaphy [8] because they generate favorable tensile strength and durability after [3, 4]. However, studies have shown that the suture material can result in increased inflammation and fibrosis at the nerve anastomosis, resulting in nerve tissue damage and a functionally poor outcome [3, 4, 9]. An established procedure to reduce suture-associated complications is the use of tissue adhesives in nerve repair [3, 4, 9–11]. One of the most commonly used alternatives for neurorrhaphy is fibrin glue [10]. Fibrin glues have been used for hemostasis and tissue adhesion since the 1940s. The adherent and hemostatic effect is based on the use of cascades of blood coagulation, in which thrombin converts fibrinogen to fibrin monomers, which crosslink covalently [4, 10]. In 1990, an animal study showed that although there was comparable tensile strength between adhesive and suture repairs, there was a trend toward improved nerve conduction velocity and higher prevalence of myelinated axons in the neurorrhaphy by suture [12]. In addition, dehiscence rates of 13% were reported in the fibrin group in this study, and dehiscence rates of up to 20% have been reported in the literature using fibrin glue in comparable studies, indicating inferiority of fibrin glue over conventional suture repair [12, 13]. In contrast to these studies with a high rate of dehiscence [12, 13], more modern fibrin glues contain formulations that include an antifibrinolytic agent that increases tensile strength [10, 14]. Despite reduced dehiscence rates of about 12.5%, modern fibrin adhesives still prove to be worse in burst strength testing and overall inferior to nerve suture repairs [14].

Cyanoacrylates were originally used as pure tissue adhesives [15] or for embolization of vessels [16] in the past and are increasingly coming into the light of science as an alternative to fibrin glue and suture material due to their ease of use and high tensile strength [17]. Upon contact with basic substances, these synthetic adhesives polymerize [10]. A disadvantage of cyanoacrylates with short alkyl chains that is frequently described in the literature is the formation of toxic metabolites such as formaldehyde and cyanoacetate [18, 19]. More recently, the formulations of cyanoacrylates have been changed to longer alkyl chains because these toxic metabolites are formed more slowly, allowing the organism to metabolize them effectively [10]. Accordingly, longer chain cyanoacrylates such as octyl cyanoacrylates are enjoying increasing popularity and cyanoacrylates have been compared to classical suture in several rat studies on sciatic nerves and no difference was found in terms of functional outcome [20, 21].

Studies evaluating the tensile strength of cyanoacrylates show contradictory results: On the one hand, octyl cyanoacrylates have been shown to have a tensile strength comparable to that of 5–0 monofilament nylon [22]. On the other hand, longer-chain cyanoacrylates have been described to be weaker than suture nerve anastomoses [23].

Recently, the novel polyurethane-based tissue adhesive VIVO has been described in the literature, which has shown a good bond between the adhesive and the tissue at the histological level in several studies [24–27] and in mechanical tests of tensile strength [28]. When applied to microvascular anastomoses, this polyurethane-based tissue adhesive showed a promising tensile strength of 1.33 [N] on average [28]. In several animal studies, VIVO showed sufficient stability over a short period of time [24, 25, 27] as well as over long-term application [26, 29]. In contrast to cyanoacrylates, significantly lower inflammatory reactions have been described for anastomoses with the biodegradable polyurethane-based adhesive compared to anastomoses with sutures [30].

One approach to quantify the biomechanical properties of neurorrhaphy methods is use of ex vivo models [6]. Experimental analysis of mechanical force by tensile testing are established in vitro methods for evaluating new neurorrhaphy methods [6, 31] before testing these methods for function in an animal model [6]. In this study, we propose an alternative anastomotic technique, which is created suture-free using the novel biodegradable tissue adhesive VIVO. This adhesive technique was compared with other suture-free adhesive techniques using fibrin glue and cyanoacrylate, as well as with interrupted sutures and native nerves. Testing was performed in an ex vivo chicken model on isolated femoral nerves. The aim of this ex vivo study was to evaluate the material and mechanical properties of sutureless adhesive-based neurorrhaphy with the gold standard of sutured nerve repair in a stress measurement.

## Methods

A total of 60 femoral nerves were used in this study and all surgical procedures were performed by a single, experienced person. After dissection of the nerve, neurorrhaphies were performed using fibrin glue (Tisseel) (*n* = 15), n-butyl-2-cyanoacrylate glue (Braun) (*n* = 15) and the novel polyurethane-based adhesive (VIVO) (*n* = 15). These procedures were then compared to the gold standard of neurorrhaphy, by four interrupted sutures (*n* = 15). Tensile force experiments were conducted on all nerve segments. The characteristics of the femoral nerves for all groups and tests are summarized in Table 1.

**Table 1:** General data for the number of samples of the performed neurorrhaphies

Groups	Chicken nerve (*n* = 60)	External diameter of nerves (mm) Mean ± SD (*n* = 60)	Time neurorrhaphy procedure (min) Mean ± SD (*n* = 60)
Suture (*n* = 15)	Femoral nerve	2.79 ± 0.29	5:06 ± 0:26
VIVO (*n* = 15)	Femoral nerve	3.03 ± 0.17	1:51 ± 0:07
Fibrin glue (*n* = 15)	Femoral nerve	2.95 ± 0.2	2:22 ± 0:11
Histoacryl glue (*n* = 15)	Femoral nerve	3.03 ± 0.1	1:28 ± 0:06

The external diameter of the nerves and the time for proceeding each neurorrhaphy are presented as mean values. *SD* standard deviation

### Femoral nerve

**Fig. 1:**
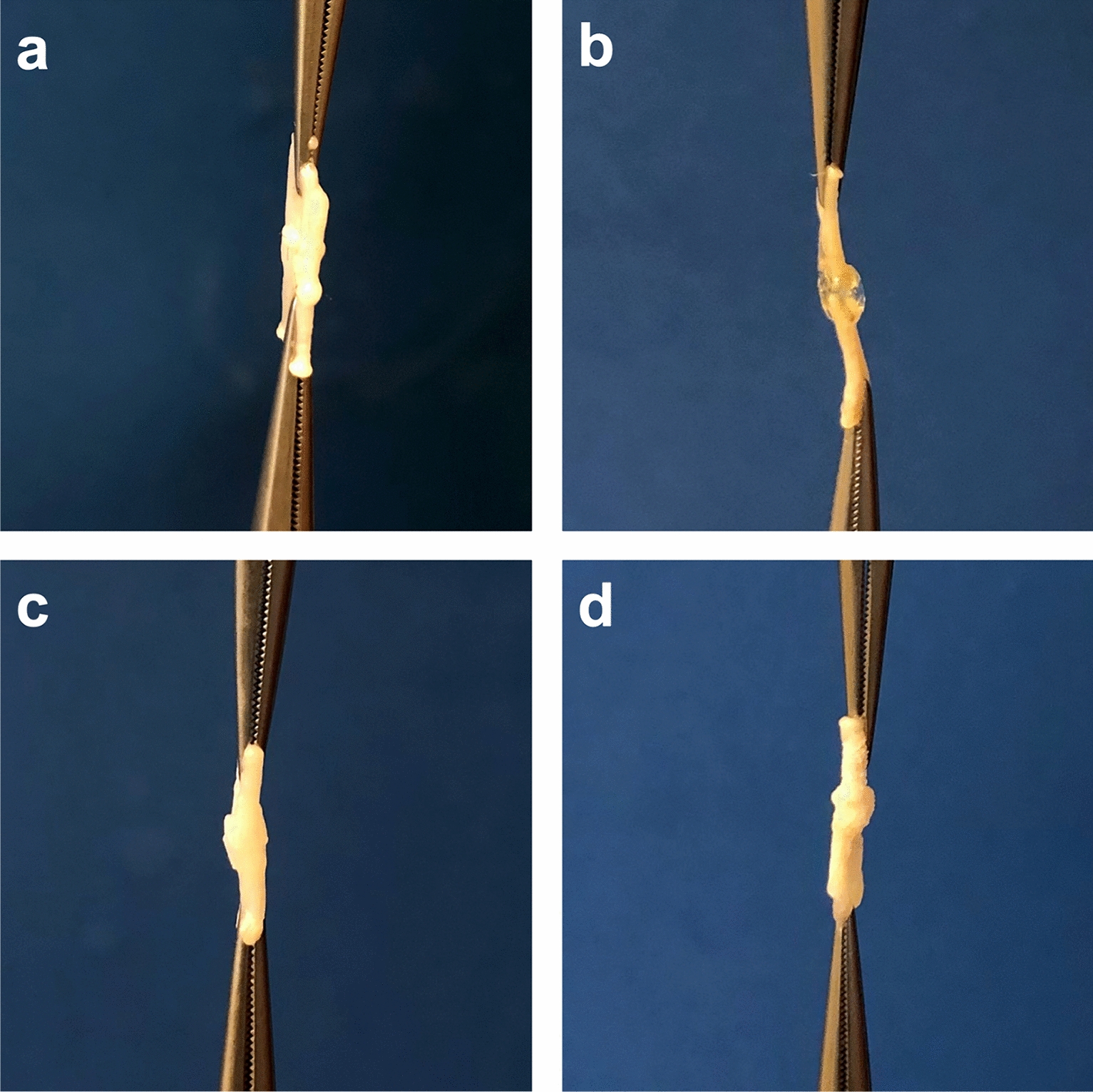
Photographs of the different neurorrhaphies: **a** suture anastomosis; **b** VIVO anastomosis; **c** fibrin glue anastomosis; **d** Histoacryl anastomosis

The use of femoral nerves provides an established model for testing new neurorrhaphy methods [6, 14, 20, 31]. Fresh male chicken legs were obtained from a local slaughterhouse within 10 min after death and transported in a modified Krebs–Ringer bicarbonate solution at 4 °C according to established protocols [28, 32]. Subsequently, according to an established protocol an incision was made on the chicken thigh above the femoral neurovascular bundles after removing of the skin [33]. Then the femoral nerves were gently dissected from femoral vessels and nerves were harvested on chilled dissection tables using magnification (Carl Zeiss Meditec AG surgical microscope, Jena, Germany) and transferred after dissection to fresh cold modified Krebs buffer solution [28, 34]. The buffer was replaced every 10 min. The nerves of the 30 chicken legs were completely dissected out and to ensure comparability of the nerves, the nerves were then dissected into segments with a length of 30 mm. (Table 1). The nerve segments were randomized into five groups: Nerves in the suture-based group (Suture) were anastomosed after specimens were halved in the middle perpendicular to the central axis and readapted with 4 microepineurial single sutures of Ethilon 10/0 (Ethicon, Hamburg, Germany). For the adhesive-based neurorrhaphy, the ends of the nerve segments were carefully adapted without gaps. The liquid tissue adhesives fibrin glue (Tisseel ®, Baxter Healthcare Corp, Deerfield, USA), N-butyl-2-cyanoacrylate glue (Histoacryl ®, Braun, Melsungen; Germany), and the polyurethane-based adhesive VIVO (VIVO, Adhesys Medical GmbH, Aachen, Germany) were first applied to the outside of the adapted nerve stumps in a two-sided, single-sided manner. After the respective adhesives were cured, the nerves were turned over and another adhesive application was made on the back side (Fig. 1). All nerves were tested immediately after preparation, and the time required to complete the neurorrhaphy was recorded.

### Uniaxial tensile test

**Fig. 2:**
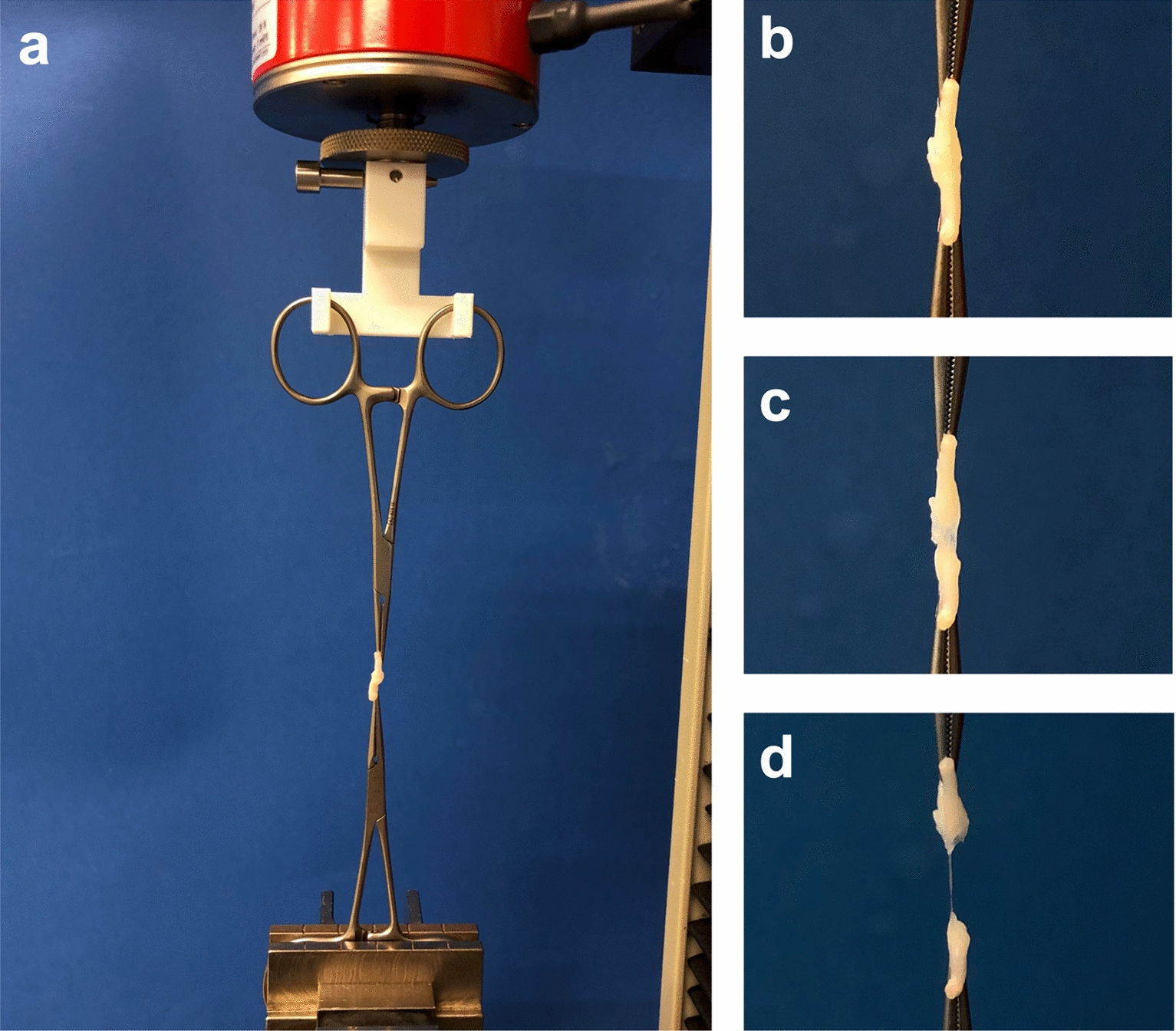
**a** Overview image of the experimental setup. The two nerve segments are connected by suture, VIVO, fibrin glue or Histoacryl and clamped in the traction machine by two arterial clamps. **b-d** Exemplary photographs of the tensile test procedure with a neurorrhaphy by fibrin glue. **b** At the beginning the nerve is clamped without tension. **c** As the traction force increases, the nerve lengthens and the first connections of the neurorrhaphy resolve under the load. **d** Complete separation of the neurorrhaphy and end point of the measurement

Fifteen nerves from each group were tested for their longitudinal tensile strength using a standard tensile testing machine (Zwick Z2.5, Zwick GmbH & Co, Ulm, Germany). The tests were conducted according to established protocols under normal climate conditions in compliance with DIN EN ISO 139. Air humidity was controlled at 65%, and a constant room temperature of 20 °C was maintained [28, 35]. After completion of the neurorrhaphy, the samples were tested directly and without intermediate storage. As previously described all samples were loaded perpendicular [28, 36] and moistened with Krebs buffer solution and nerve ends were mounted in arterial clamps fixed at the top and bottom. The upper artery clamp was suspended in a fixture constructed specifically for this experiment using 3D printing, and the lower clamp was clamped in the jaws of the tensile testing machine. The specimens were clamped in a neutral position without preload (Fig. 2). According to an established protocol, load-to-failure curves were generated by loading the nerves under position control at 20 mm per minute until failure [35]. Each nerve testing was graphically plotted, and two endpoints were determined. L_0_ of specimen was when load exceeded 0 [cN]. The standard force at failure of the nerve anastomosis was determined from computer-based data and represents the apex in the generated curves. In addition, the extension △L/L_0_ in percent at the time of peak force was determined. Subsequently, the type of failure, including suture pullout and adhesion or adhesive breakage, was determined by microscopic inspection.

### Statistical analysis

All data were evaluated using GraphPad Prism 7.0 (GraphPad Software, San Diego, USA). Parametric statistics were applied with data that passed the Kolmogorov–Smirnov test. Relevant results were analyzed by one-way analysis of variance (ANOVA) for multiple comparisons followed by Tukey`s post hoc analysis. The standard force and time for neurorrhaphy was analyzed with Kruskal–Wallis test for nonparametric independent variables to compare the differences between parameters. All data represent the means ± SD. Statistical significance was determined when *p* ≤ 0.05.

## Results

**Fig. 3:**
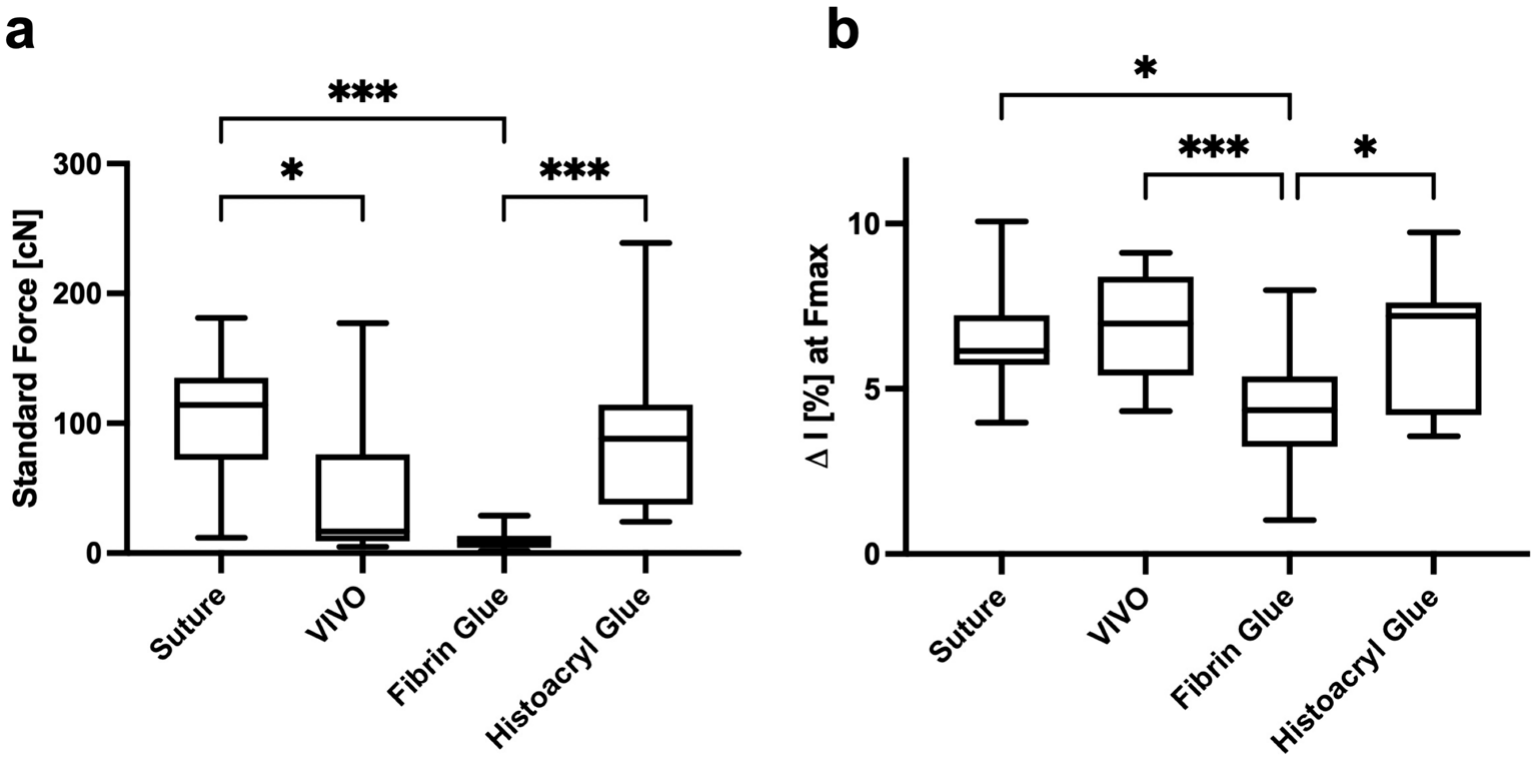
Box and whisker graphics of maximal tensile force and △L/L_0_ of all groups. **a** There is no statistical difference between maximal tensile force of Suture group and Histoacryl group. Both groups have the highest tensile force, followed by the VIVO group. Neurorrhaphy using fibrin glue showed the lowest tensile forces.** b** Comparison of the △L/L_0_ of all groups shows similar trends, with the VIVO group showing the greatest flexibility during maximum traction. ***p ≤ 0.001; *p ≤ 0.05. All scatter plots represent the means ± SD

The used femoral nerves had a comparable circumference of 2.79 ± 0.29 [mm] in the suture group, 3.03 ± 0.17 [mm] in the VIVO group, 2.95 ± 0.2 [mm] in the fibrin glue group and 3.03 ± 0.16 [mm] in the Histoacryl group. The time to create the neurorrhaphy showed the longest duration in the suture group with a time of 5:06 ± 0:26 [min], where, on the other hand, the adhesive-based neurorrhaphies with Histoacryl and VIVO could be created significantly faster (*p* < 0.001). The fastest anastomosis was created with 1:28 ± 0:06 [min] in the Histoacryl group with no difference to VIVO with 1:51 ± 0:07 [min]. Nerve anastomoses using fibrin glue, with 2:22 ± 0:11 [min] also took significantly more time compared with the use of Histoacryl (*p* < 0.001) Table 1. Table 2 and Fig. 3 show the tensile strength testing of the four groups. In the fibrin glue group, one nerve anastomosis was destroyed during the clamping process, so only 14 nerves could be tested for tensile strength in this group. The suture group showed the highest values during tensile strength determination. The applied forces rose steeply to a global maximum of 100.2 ± 47.24 [cN] at an elongation of 6.41 ± 1.45 [%]. Thus, the suture group has significantly higher tensile strength compared to the neurorrhaphy using VIVO 45.47 ± 50.7 [cN] (p = 0.019) and the fibrin glue group, with 10.55 ± 7.33 [cN] (p < 0.001). There is no significant difference in tensile strength between suture and Histoacryl group (Fig. 3). Neurorrhaphies based on Histoacryl glue demonstrated tensile strength of 91.05 ± 56.95 [cN] at an extension of 6.34 ± 1.96 [%]. Compared to the neurorrhaphy procedure using fibrin glue, VIVO with a △L/L_0_ of 6.96 ± 1.58 [%] showed a highly significant higher elongation at the maximum load (*p* < 0.001). Likewise, the neurorrhaphy procedures using suture (*p* = 0.01) and Histoacryl glue (*p* = 0.014) showed significantly higher elongation compared to the sutured nerves at maximal loading.

**Table 2:** Examination parameters of the tensile tests

Groups	Tensile force [cN] Mean ± SD	Nerve rupture	Suture pullout	Adhesion or adhesive breakage	△I (%) at Fmax Mean ± SD
Suture (*n* = 15)	100.2 ± 47.24	8/15	2/15	N.A	6.41 ± 1.45
VIVO (*n* = 15)	45.47 ± 50.7	2/15	N.A	13/15	6.96 ± 1.58
Fibrin glue (*n* = 14)	10.55 ± 7.33	1/14	N.A	13/14	4.38 ± 1.66
Histoacryl glue (*n* = 15)	91.05 ± 56.95	2/15	N.A	13/15	6.34 ± 1.96

Tensile force [cN] = results of the tensile test; Nerve rupture = number of ruptured nerves outside the applied neurorrhaphy; Suture pullout = sutures that were pulled out of the tested nerves instead of breaking; Adhesion or adhesion break = breakage of the adhesive in the area of the neurorrhaphy; the data of the tensile force [cN] and △I(%) are presented as mean values. SD = standard deviation. N.A. = not applicable

**Fig. 4:**
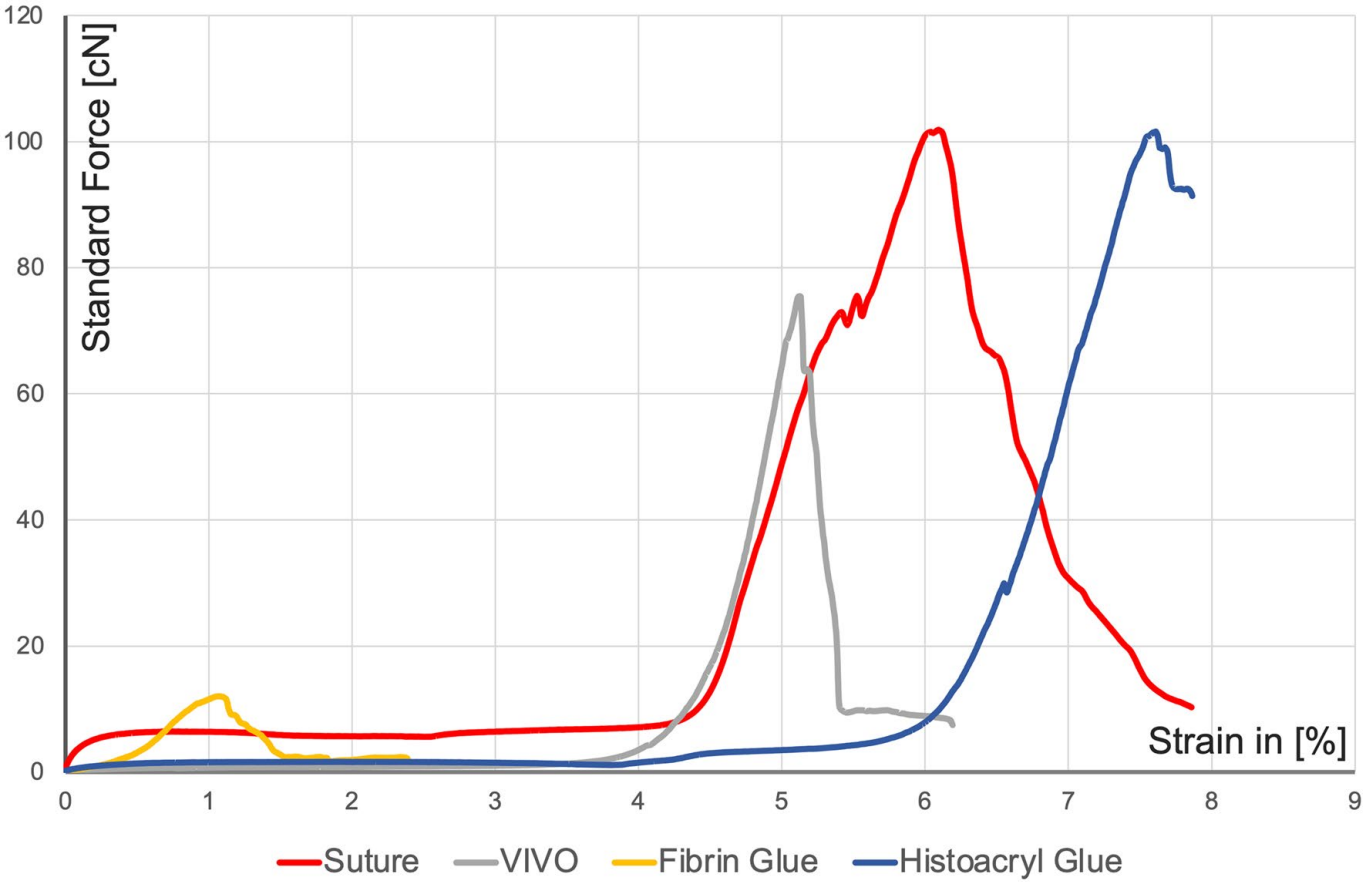
Graphical illustrations of axial forces of characteristic specimens

Representative curves of the tensile tests of the respective groups can be seen in Fig. 4. A representative neurorrhaphy of Histoacryl glue showed a constant increase up to a local maximum of 101.67 [cN] at an extension of 6.49 [%]. After this maximum, there was an almost continuous drop in force.

## Discussion

For over 80 years, neurorrhaphy using microsutures has been the gold standard for nerve reconstruction. However, this technique has limitations in terms of inflammation and scarring [3, 4], which negatively affects the regeneration of peripheral nerves [37]. Therefore, there is a need to investigate alternative repair methods that are comparable to or better than the microepineural suture method. In addition, microsurgical repair using microsuture coaptations requires great manual dexterity as well as extensive microsurgical experience [38] which is reflected in a longer operation time. In this study, it was shown that the microneurosurgical technique using four single sutures by an experienced surgeon has an average duration of 5:06 [min]. Koulaxouzidis et al. [9] and Whitlock et al. [38] both reported significant acceleration of the procedure by using adhesives in the creation of a neurorrhaphy. Accordingly, the microneurosurgical procedure was significantly shortened by an average of 3:15 min using Histoacryl adhesive and by an average of 3:38 min using VIVO tissue adhesive compared with microepineurial suture repair. Contrary to what is reported in the literature [9, 38], no significant acceleration of neurorrhaphy could be obtained by using fibrin glue compared to the suture group, which can be explained by the faster setting time of the cyanoacrylate Histoacryl or the polyurethane-based adhesive VIVO. In addition, the literature describes that the technically simple use of tissue glue for peripheral nerve repair has the advantage that both naive and experienced surgeons can obtain the same outcome [38].

Another reason that the use of sutures has been proven to be largely successful in neurorraphy is the favorable tensile strength and durability of the nerve reconstruction [10]. In accordance with this, our results show that the sutured nerve repair has a sufficient tensile strength of 100.2 [cN].

A described disadvantage of nerve repair techniques using fibrin glue is the deficiency in tensile strength. Accordingly, this experiment shows that the fibrin glue group has tensile strength of 10.55 [cN], only about 10% of the tensile values of the nerve suture and Histoacryl groups. The relevant literature on studies evaluating the tensile strength of cyanoacrylates describe conflicting results [22, 23]. However, the tensile results of this study demonstrate that the tensile strength of the longer-chain cyanoacrylate Histoacryl with 91.05 [cN] is comparable to that of monofilament microepineural nylon sutures of strength 10–0. This is the first-ever study to use the polyurethane-based VIVO adhesive in a neurorrhaphy procedure and compare its tensile strength with other nerve reconstruction procedures. On the one hand, the data demonstrate that VIVO with a tensile strength of 45.47 [cN] is inferior to neurorrhaphy by suture. On the other hand, it illustrates that the tensile strength of VIVO is approximately four times the tensile strength of fibrin glue, which represents the most commonly used neural regeneration procedure after suture [10]. The nerve repairs using VIVO and Histoacryl glue showed larger variations in the standard deviations of ± 50.7 [cN] and ± 56.95 [cN], respectively. Although both adhesives have the ability to bind in moist tissue, the authors assume that despite careful drying of the nerves before applying the adhesive, different degrees of moisture could have led to the measurement deviations.

To ensure an ideal environment for axon regeneration after repair of a peripheral nerve, internal and external tension on the affected nerves should be avoided as far as possible. Therefore, immobilization of affected limbs for 2 weeks is often recommended after neurorrhaphy in the literature [39]. This applies equally to suture repair and alternative neurorrhaphy procedures [39, 40]. After 2 weeks, there is the same risk of rupture of nerve repairs with sutures or adhesives [4]. One limitation of our in vitro study is the fact that only axial tensile loads were tested, which means that the influence and force load on the repaired nerve caused by movement of one limb can only be analyzed to a limited extent. In addition, the question of functional regeneration and the healing process of a damaged nerve remains unanswered, so that further in vivo analyses in animal models are required.

In addition to tensile strength, another requirement for an anastomosis is sufficient elasticity [27]. An ex vivo tensile strength study describes a favorable elongation of VIVO in vascular anastomoses [28]. Our results with a △L/L_0_ of 6.96 [%] and the finding of the strongest elongation of all groups reinforce these results. Here, further in vivo studies are essential to investigate the stability and elongation of neurorrhaphy using VIVO compared to neurorepair using fibrin glue and cyanoacrylate adhesive in a living organism after several weeks. Critical to this study is the fact that all nerve anastomoses were created with adhesives without the use of adaptive sutures. In clinical application, depending on the nerve, sutures of the epineurium, perineurium, and fascicles may be considered to allow adaptation of the nerve in the correct sequence. On the contrary, according to Chow et al. [10], several studies indicate that augmentation of the traditional suture method with, for example, fibrin glue does not generate any improvement in nerve conduction velocities, motor action potentials, or axonal regeneration compared with the suture technique alone. In addition, a systematic review of 16 different studies demonstrated comparable conduction velocities and benefits in terms of reduced granulomatous inflammation, better fascicle alignment, and better axonal regeneration when compared to sutured nerve repairs alone using fibrin glue exclusively during neurorrhaphy procedure [41]. For these reasons and the possibility of an isolated study of the adhesive strength of a tissue adhesive, the authors decided against supportive sutures in neurorrhaphy.

Furthermore, studies have shown that nerve repair using sutures can result in increased inflammation and fibrosis at the coaptation site [3, 4, 9]. These inflammatory processes, in turn, lead to tissue damage and poor functional neural outcomes [3, 4, 9]. In the search for less complicating alternatives, adhesives have been increasingly considered in securing nerve repairs [10]. Although cyanoacrylates are produced with formulations of longer alkyl chains, an existing disadvantages of these adhesives is the release of small amounts of toxic metabolites [10]. In contrast, the polyurethane adhesive VIVO has been described in the literature to elicit a physiological, non-harmful tissue–biomaterial interaction in histological evaluations [27]. Furthermore, in several studies with microvascular anastomoses, a good and less inflammatory tissue reaction towards the suture material was determined [24, 30]. Furthermore, for clinical application, comparative findings through long-term studies on stability in the living organism, tissue interactions, toxicity as well as immune response by VIVO, fibrin glue and cyanoacrylate versus neurorrhaphy by suture are critical points. Therefore, future short-term and long-term animal studies are essential addressing these parameters as well as the biocompatibility of the tissue adhesives.

Spotnitz and Burks describe that the approval of medical adhesives by regulatory authorities in particular is a limiting factor for the application and research of these adhesives in clinical use [42]. In humans, the N-butyl-2-cyanoacrylate glue used in this study is currently only approved for use on external body surfaces [42], and investigations according to peripheral nerve repair were only conducted in animal models [10]. The novel polyurethane adhesive VIVO, on the other hand, is not yet approved for use in humans, so that currently only animal tests could be carried out with this tissue adhesive [24–27, 29, 30, 43]. Although fibrin adhesives have long been approved for various medical applications, the data available on their use for the repair of peripheral nerves in humans are very limited [10]. Given the lack of objective data and or clinical applications of adhesives in peripheral nerve repair, the analysis of the efficacy of these techniques in humans remains largely unanswered. On the other hand, human cadaver studies offer the potential to analyze adhesives in human tissue even without approval for clinical use. In our study, nerves from the chicken were used, which only represent a model for biomechanical analysis and are inferior to utilization of real human tissue.

## Conclusions

Within the limiting background of an in vitro study, it could be shown that sutureless neurorrhaphy using Histoacryl and VIVO achieved very promising results in terms of traction and proved superior to sutured nerve repairs in terms of shortened procedure time as well as easier handling. In addition to sufficient tensile strength, the elongation of VIVO represents favorable properties for a neurorrhaphy procedure. Despite easier handling, sutureless neurorrhaphy with fibrin glue demonstrates to be inferior to the other procedures. Future comparative short- and long-term in vivo studies are needed to evaluate the tensile strength and biocompatibility of Histoacryl and VIVO for neurorrhaphy and to validate neural outcomes and immunologic responses of a living organism.

## Data Availability

All data generated or analyzed during this study are included in this published article.
